# Endoscopic Transmural Therapy of Pancreatic Fistulas in an Interdisciplinary Setting—A Retrospective Data Analysis

**DOI:** 10.3390/jcm12134531

**Published:** 2023-07-06

**Authors:** Clara Meierhofer, Reinhold Fuegger, Georg O. Spaun, Helwig Valentin Wundsam, Patrick Kirchweger, Matthias Biebl, Rainer Schoefl

**Affiliations:** 1 Department of Gastroenterology, Ordensklinikum Barmherzige Schwestern Linz, 4010 Linz, Austria; 2 Department of Surgery, Ordensklinikum Linz Barmherzige Schwestern, 4010 Linz, Austria

**Keywords:** pancreas, fistula, surgery, interventional endoscopy, transmural drainage

## Abstract

Pancreatic fistulas belong to the most feared complications after surgery on or near the pancreas, abdominal trauma, or severe pancreatitis. The majority occur in the setting of operative interventions and are called postoperative pancreatic fistulas (POPF). They can lead to various complications, including abscesses, delayed gastric emptying or hemorrhages with a significant impact on morbidity and mortality. Several risk factors have been identified, including smoking, high BMI, male gender, and age. Prophylactic measures and treatment options have been explored but with limited success. This study aimed to analyze the incidence and management of pancreatic fistulas treated in a tertiary referral center, particularly focusing on an endoscopic approach. The data of 60 patients with clinically relevant pancreatic fistulas were analyzed between 2018 and 2021. Different treatment approaches, including conservative management, percutaneous drainage, transpapillary stenting, and endoscopic transmural drainage, were evaluated. An endoscopic transmural approach using lumen-apposing metal stents (LAMS) was used in almost half of this cohort showing promising results, with a high rate of fistula closure in refractory cases and a mean time until closure of 2.7 months. The findings suggest that an endoscopic approach, particularly using LAMS, can be effective in the management of pancreatic fistulas.

## 1. Introduction

Pancreatic fistulas belong to the most feared complications after surgery on or near the pancreas, abdominal trauma, or severe pancreatitis. The majority occur in the setting of operative interventions and are called postoperative pancreatic fistulas (POPF) with rates that vary widely from 2% to well over 20% [1].

According to the definition of the International Study Group for Pancreatic Fistula (ISGPF) they originate from a leak of the pancreatic ductal system into and around the pancreas. Diagnosis is established when peritoneal drainage fluid can be measured, in which the amylase concentration exceeds the upper limit of normal serum amylase level by three times or more than 300 IU/L [1]. A variety of complications can follow such as intra-abdominal abscess, delayed gastric emptying, and postoperative hemorrhage as the leakage of pancreatic juice can lead to vessel erosion. Systemic inflammation may result in sepsis or organ failure with an increased rate of perioperative mortality [2]. The ISGPF proposed a grading system in which POPF are classified according to their severity. According to the last update in 2016, grade A POPF are classified as biochemical pancreatic fistulas. They have no clinical impact on the postoperative course and no further treatment is required. In contrast, grade B and C pancreatic fistulas have an impact on the clinical course and result in a relevant change of management with need for interventions, reoperations, and intensive care therapy. These clinically relevant pancreatic fistulas (CR-POPF) are associated with an extended hospital stay and an increased risk for mortality up to 20% [2].

So far, smoking, high BMI, male gender and age were identified to increase the risk whereas diabetes mellitus seems to reduce it [3]. Concerning pancreaticoduodenectomy, a small pancreatic duct with a diameter ≤ 3 mm and soft pancreatic tissue have been identified to be the most important risk factors for postoperative pancreatic fistulas [4]. Other surgery-related factors that have been described include a prolonged operation time, excessive blood loss, the non-performance of ligation of the main pancreatic duct, transection at the tail and additional organ resection [5]. Distal pancreatectomy seems to have a higher incidence of POPF as compared to pancreaticoduodenectomy. At least partly, this could be explained by the preservation of the Oddi sphincter, which may lead to a higher intrapancreatic duct pressure following distal resection [3].

Taking the morbidity of a clinically relevant fistula into account, prophylactic measurements and treatment options have become a hot topic in recent years. In pancreatoduodenectomy, fibrin sealants or omental wrapping did not achieve consistent results regarding the incidence of POPF [5,6,7]. In distal pancreatectomy, different stump closure methods (stapler vs. hand-sewn), reinforcement of the resection margin with an absorbable fibrin sealant or a teres ligament patch and minimally invasive techniques such as a laparoscopic or robotic-assisted approach did not show a significant reduction [6,8,9]. The routine use of intraperitoneal drain placement allows the evacuation of blood, pancreatic juice, bile, and lymphatic fluid. However, the time of removal, number of inserted drains or even if an omission of drainage is possible, remains unclear. There seems to be a difference between pancreatoduodenectomy and DP, keeping in mind that a POPF after pancreatoduodenectomy can originate from an underlying anastomotic dehiscence. The optimal timing of drain removal is discussed controversially in the available literature. When looking at the existing data, the lack of an accurate definition of early drain removal seems to be predominant. Some studies suggest that early drain removal decreases the risk of POPF but since periprocedural treatment varies between these studies, the optimal timing remains unclear.

Management of clinically relevant fistulas should be discussed across disciplines and be based on the updated ISPGPF grading [4]. Primary percutaneous catheter drainage is often the first intervention for CR-POPF in the absence of organ failure. Given its minimally invasive nature, percutaneous drainage results in a fistula closure rate of 77.1% [10]. Furthermore, endoscopic approaches such as transpapillary stent placements across leakages or sphincterotomy to decrease the pressure gradient at the pancreatic sphincter could be considered [11]. As EUS has become widely available, transmural stenting has started to influence the management of peripancreatic fluid collections (PFCs). The role of EUS-guided drainage has evolved from plastic stents to fully covered self-expandable metal stents (fcSEMS) and, recently, lumen-apposing metal stents (LAMS). These LAMS provide a single-step delivery platform guided by endoscopic ultrasound. They can be applied in various areas, including drainage of fluid collections, decompression of obstructed ductal systems, and to create connections between organs for further endoscopic interventions if needed. Another advantage of LAMS is their ability to closely approximate adjacent structures, reducing the risk of leaks and perforations. However, a thorough understanding of the technical aspects of LAMS placement is crucial to minimize complications and enhance clinical outcomes. Nonetheless, studies mostly focused on fluid collections following acute pancreatitis and walled off necrosis, whereas data on its use in the management of CR-POPF are rare. Surgical interventions for the treatment of pancreatic fistulas decreased continuously during the past decade, being reserved for patients who are not candidates for a minimally invasive intervention or whose condition is progressively worsening with catheter drainage. There is no agreement on how to use pharmacological treatments such as somatostatin analogues when fistulas are already present. The recently published Guidelines on the treatment of pancreatic cancer in Germany do, however, propose a periprocedural somatostatin treatment in high-risk situations referring to a meta-analysis that showed a reduction in frequency and complications of POPFs [12]. Adequate intensive care support, including enteral nutrition and antibiotic therapy, are mandatory in patients with organ failure and infection [13].

This study aimed to investigate the incidence and treatment options for patients suffering from pancreatic fistulas in an expert center. We analyzed the fistula closure rate and the time to fistula closure of the applied therapeutic measures, focusing especially on an endoscopic approach.

## 2. Patients and Methods

We retrospectively analyzed the data of patients treated with clinically relevant pancreatic fistulas at the Department of Surgery and the Department of Gastroenterology and Hepatology over the last 4 years. Demographic and clinical characteristics are summarized in Table 1.

Overall, 60 patients with pancreatic fistulas of various origins were evaluated. The composition of our patient cohort represents a selection according to our focus on pancreatic surgery and endoscopy. Data of patients with postoperative pancreatic fistula were drawn from the prospective pancreas registry that is conducted by the surgical department. A total of 180 pancreaticoduodenectomies and 112 distal (left-sided) pancreatectomies were performed in our center between 2018 and 2021 with an incidence of 119 POPF (40.8%). According to the 2016 updated ISGPF recommendations, 74 cases corresponded to biochemical leaks (62.2%), 40 to Grade B (33.6%) and 5 fistulas were classified as Grade C (4.2% of all POPF). When focusing on the clinically relevant POPF, a total of 45 fistulas were observed resulting in a CR-POPF rate of 15.4%. POPF-associated mortality was low, ranging from 1.7% in the first 30 days to 2.5% after 90 days.

Diagnosis of surgical patients included 17 adenocarcinomas of the pancreas, 9 pancreatic neuroendocrine tumors (pNET), three adenocarcinomas of the duodenum, 5 gastric cancers, 7 premalignant cystic lesions, and four patients who suffered from pancreatic fistulas after cytoreductive surgery due to ovarian carcinomas. One patient suffered from a POPF after a laparoscopic biopsy from the pancreas to help diagnose an autoimmune pancreatitis type I (Table 2).

Only 7 (11.6%) were diagnosed with PF after necrotizing pancreatitis and 4 (6.7%) in the setting of a chronic pancreatitis. There were 3 fistulas documented after trauma to the abdomen (5%).

Peripancreatic drainage was applied in all patients undergoing pancreatic resection. For the diagnosis of postoperative pancreatic fistulas, amylase concentrations were routinely measured in the drainage fluid on postoperative days 1 to 3. Pancreatic fistulas were defined as an amylase concentration in the drainage fluid more than 3-fold the upper limit of the normal serum amylase level and graded according to the 2016 updated recommendations of the International Study Group on Pancreatic Fistula (ISGPF) [1]. Grade A corresponds to biochemical evidence of a fistula without clinical consequence (biochemical leakage). Grade B fistulas required persistent drainage over 3 weeks, percutaneous or endoscopic drainage, an angiographic procedure for bleeding, or signs of infection without organ failure and grade C fistulas result in reoperation, organ failure, or death.

Interventions for the treatment of pancreatic fistulas were conservative management with prolonged surgical drainage or secondary percutaneous drainage, transpapillary stenting, endoscopic transmural stenting, or a combination of these procedures. Transmural stenting was predominately performed using LAMS (The Hot AXIOS stent, Boston Scientific Corporation, Natick, MA, USA). These lumen-apposing metal stents were placed within minutes using a curvilinear echoendoscope. After positioning the tip of the endoscope, the intestinal wall and the targeted area were punctured by applying cautery. Next, the distal flange of the LAMS was released, which can be monitored on ultrasonography. After positioning of the catheter, the upper stent deployment portion was unlocked, and the proximal flange of the LAMS was deployed either under endoscopic view or by pushing it out through the working channel of the echoendoscope during withdrawal. Different saddle lengths and lumen diameters are available, facilitating the adjustment based on the clinical presentation. In this data set, mainly stents with a saddle length of 10mm and a lumen diameter of 8mm were used.

Prophylactic transpapillary stenting was not routinely used before distal pancreatectomy. Only one patient with a preoperative high fistula risk received a prophylactic transpapillary stenting without developing a POPF after surgery and therefore is not part of this data collective. The primary outcome parameter was the rate of fistula closure when using an endoscopic transmural approach. Furthermore, time until fistula closure was analyzed comparing the effect of percutaneous drainage, endoscopic transmural drainage and transpapillary stenting.

## 3. Results

As far as treatment is concerned, a total of 60 pancreatic fistulas were managed. All fistulas included in this analysis were graded as clinically relevant, 53 fistulas corresponded to Grade B and 7 were classified as Grade C. In surgical patients diagnosed with POPF during the initial stay, an intraoperatively positioned drainage was maintained at the time of discharge. The therapeutic strategy is summarized in Table 3.

In detail, a total of 22 patients (36.6%) had a resolution of pancreatic fistulas with conservative management including a secondary percutaneous drainage within 29 days (median, IQR 13–58). Another 13 cases (21.7%) were treated simultaneously with transpapillary pancreatic stenting, as shown in Table 4. When transpapillary stenting was started, PCD was either removed at the time of stent placement or two to four weeks after the procedure. Transpapillary stents were removed 12 weeks after ERCP.

This data should be interpreted with caution because they only represent 58.3% of the fistulas treated in this cohort excluding those that needed an additional transmural approach or rescue surgery. Furthermore, transpapillary stents were kept in place for 12 weeks regardless of fistula closure, making an adequate documentation of time until resolution not possible in this retrospective data analysis. In total, 38 fistulas (63.3%) needed more than one treatment approach.

**Figure 1:**
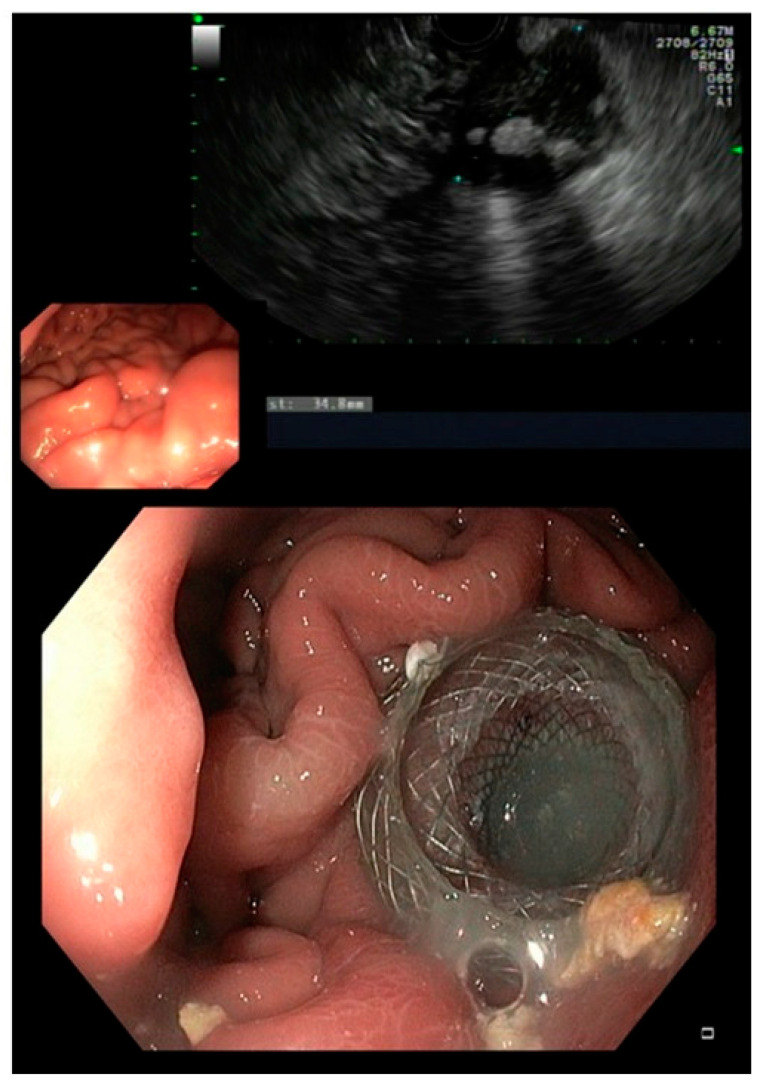
EUS showing a fluid collection and endoscopic view on a LAMS.

In our cohort, an endoscopic transmural approach was chosen in 25 patients (41.7% of all documented fistulas) of whom one patient also received a somatostatin analogue postoperatively. EUS-guided drainage using LAMS was the treatment of choice, whereas two patients received transmural drainage using only double-pigtail stents (Figure 1).

**Figure 2:**
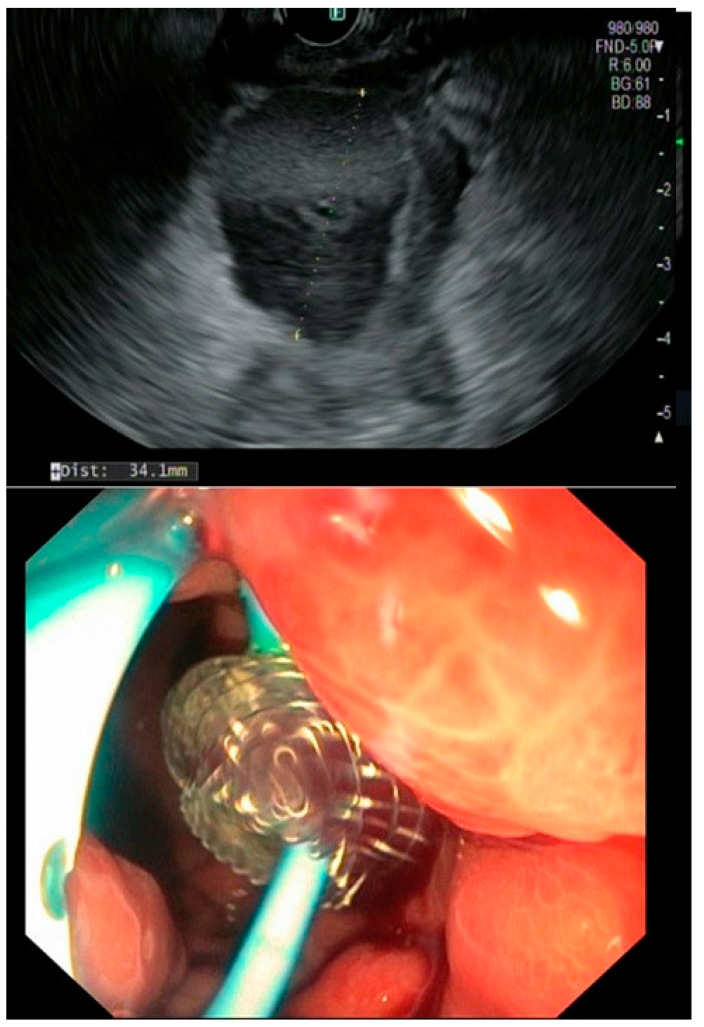
EUS showing a fluid collection and endoscopic view on a LAMS plus a double pigtail.

Only in four patients, the abdominal drain inserted intraoperatively, were removed before the onset of symptoms (in average four weeks). The remaining patients already underwent PCD before an EUS-guided drainage was approached. When transmural drainage was achieved, PCD was either removed or, in case of infection, kept in place for irrigation. Technical success was achieved in 96% owing to one case of LAMS maldeployment during EUS-guided intervention. All LAMS were removed within a period of 6–8 weeks. If needed, a double pigtail was kept in place to further facilitate a continuous drainage (Figure 2). The mean time until fistula closure was 2.7 (range 2.1–5.3) months, keeping in mind that transmural stenting was mainly approached in settings of refractory fistulas.

There was no patient treated with a somatostatin analogue alone. Three patients had to undergo total pancreatectomy because of chronic fistulation with persistent organ failure (5%). The 30-day mortality rate in the entire patient cohort was 6.7%.

Effects on fistula closure and time until fistula closure using a transmural approach are presented in Table 5.

**Figure 3:**
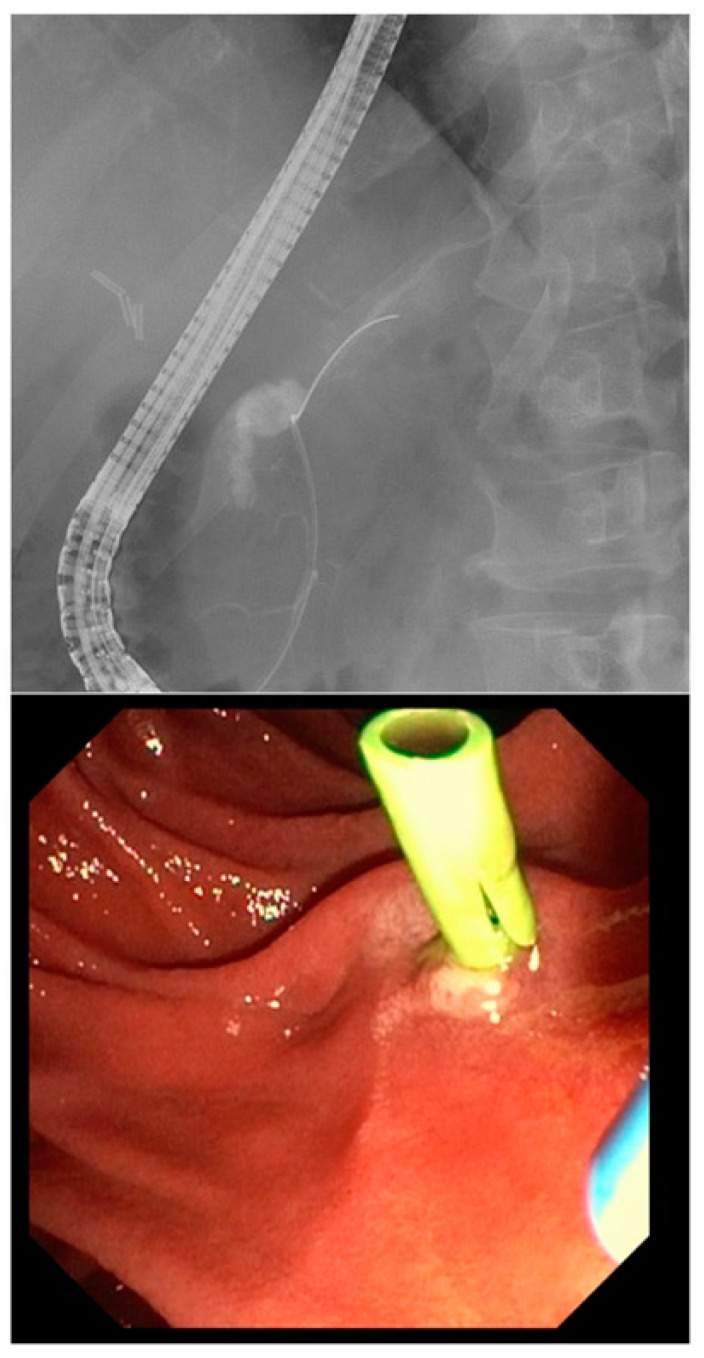
Radiograph of a pancreatic fistula during ERCP and transpapillary stenting.

In this patient cohort, the treatment strategy was changed in case of fistula persistence after 4–6 weeks following one intervention. If PCD resulted in an ongoing collection, EUS-guided transmural stenting was performed if technically possible prior to placing a transpapillary stent after sphincterotomy (in case of left-sided resection). If fluid collections were not accessible via EUS, patients received an ERCP. Pancreatic stent replacement was then performed every 3 months or, in case of resolution, removed. Short-term imaging controls helped to adjust the treatment course and EUS drainage added when possible (Figure 3).

During a follow-up period of 3 months, only the patient with the initially failed transmural drainage experienced fistula recurrence. In this setting, a transpapillary stenting after sphincterotomy was then performed and fistula closure was achieved 4 months later.

## 4. Discussion

Pancreatic fistulas remain a clinical challenge with a significant impact on morbidity and mortality. The majority of pancreatic fistulas are the consequence of surgery, especially pancreatic surgery. More rare fistulas emerge as complications of acute and chronic pancreatitis and trauma. Publications on treatment strategies for pancreatic fistulas are limited in their conclusion, probably biased by divergent patient cohorts and institutional selection, as well as referral practices. Therefore, most reports regarding therapy of pancreatic fistulas focus on specific etiology, with postoperative fistulas as the predominant subject. However, we aimed to analyze all pancreatic fistulas independently of their origin and concentrating on the applied treatment strategies.

POPF were the predominant type in our cohort, reflecting the high case volume of our pancreatic surgery center. When looking at the available literature, fistulas associated with organ failure, reoperation or death (grade C) occur with an incidence of 0–4.9% [14]. Our presented data from the prospective pancreas registry show comparable findings given a POPF C rate of 4.2%. Furthermore, our fistula-related mortality rate ranging from 1.7% within the first 30 days postoperatively to 2.5% after 90 days corresponds exactly with a meta-analysis published by Pedrazzoli et al. summarizing the therapeutic results of more than 60,000 patients with POPF [15]. In consistency with the previous literature, mortality was increased in this patient cohort of clinically relevant fistulas, that needed more intensive treatment, to 6.7%.

With respect to the surgical patients of our cohort, aside from the negative impact of CR-POPF on morbidity and survival in general, oncologic results were worsened. Adjuvant chemotherapy or radiochemotherapy was delayed or even avoided in the presence of CR-POPF, thus impairing overall and disease-free survival [16].

The most striking development regarding treatment strategies of pancreatic fistulas is the increasing use of endoscopic transmural techniques, especially LAMS. In our cohort, the use of EUS-guided drainage with LAMS has become more and more popular following the trend in interventional endoscopy. This transmural stenting approach was applied in almost half of our treated individuals with excellent technical and clinical success. However, this kind of intervention needs an experienced endoscopist and institutional expertise. While recent publications revealed good evidence on the use of LAMS in the setting of severe pancreatitis with walled off necrosis, data on its use in pancreatic fistulas and post-surgical fluid collections are still lacking, as most studies were using pigtail drainages for transmural stenting. A recently published series over 47 patients from Dongwook Oh et al. [17] on the use of LAMS in postoperative pancreatic fluid collections demonstrated a clinical success of nearly 90.2% (technically successful in 87.2%). Results of our retrospective series further support these outcomes, favoring a minimally invasive management of pancreatic fistulas using LAMS devices. Keeping these aspects in mind and considering liquid loss and tube-related complications associated with external drainage, internal transmural drainage using LAMS represents a safe and clinical successful alternative. However, questions regarding timing and optimal patient selection using LAMS remain. Furthermore, expertise and essential resources, predominantly the question of costs, determine the choice of treatment.

As far as endoscopic interventions such as sphincterotomy or stent placement across the papilla are concerned, pathophysiologic considerations followed the theory that reducing the intraductal pressure in the biliopancreatic system and in the retroperitoneum could help with fistula resolution. Some researchers underlined, that fistulas will close when the pressure gradient along the fistula approaches zero [11,18]. However, the role of sphincterotomy and transpapillary stenting in the treatment or prevention of pancreatic fistulas is discussed controversially. This is due to limited evidence, especially for fistula prevention, intervention-associated morbidity and discrepancies concerning the success rates of fistula closure. In our cohort, a transpapillary approach was predominantly chosen when conservative management resulted in ongoing peripancreatic fluid collections and not as a preemptive attempt to avoid the occurrence of pancreatic fistulas. Reviewing the recently published guidelines on the treatment of pancreatic cancer in Germany, a risk evaluation should be performed when a prophylactic intervention was considered. Assessing the individual risk of POPF as early as possible, ideally before or during the operation, could be a rational approach. Various scoring systems have been developed for this purpose, the most common of which is the Fistula Risk Score (FRS), which can be calculated intraoperatively [19]. A future strategic approach could be based on calculating a patient’s individual risk.

When considering the treatment of pancreatic fistulas in our cohort, the modality of choice was a step-up approach starting with PCD and, in case of persistence, the introduction of EUS-based interventions with or without transpapillary pancreatic duct drainage.

A meta-analysis and systematic review on this topic by Mohan et al. [10] concluded that in terms of clinical outcome, transmural drainage using EUS is a safe, effective, and preferable modality for the management of post-operative pancreatic fluid collections. Regarding technical success rates and adverse events no significant differences between endoscopic transmural and percutaneous modalities were found. However, comparisons of treatment strategies for pancreatic fistulas should be made with extreme caution, due to a wide inter-institutional divergence in patient selection and a high changeover of modalities. This is also reflected in our data using a step-up approach with a change of treatment documented in over 63.3%. Furthermore, the time until fistula closure of roughly one month for the conservative approach by a prolonged endurance of operative drains or a secondary placed PCD cannot be seriously compared with 2.7 months for fistula closure using endoscopic transmural drainage, considering that the latter was applied in persistent and recurrent fistulas. 

**Figure 4:**
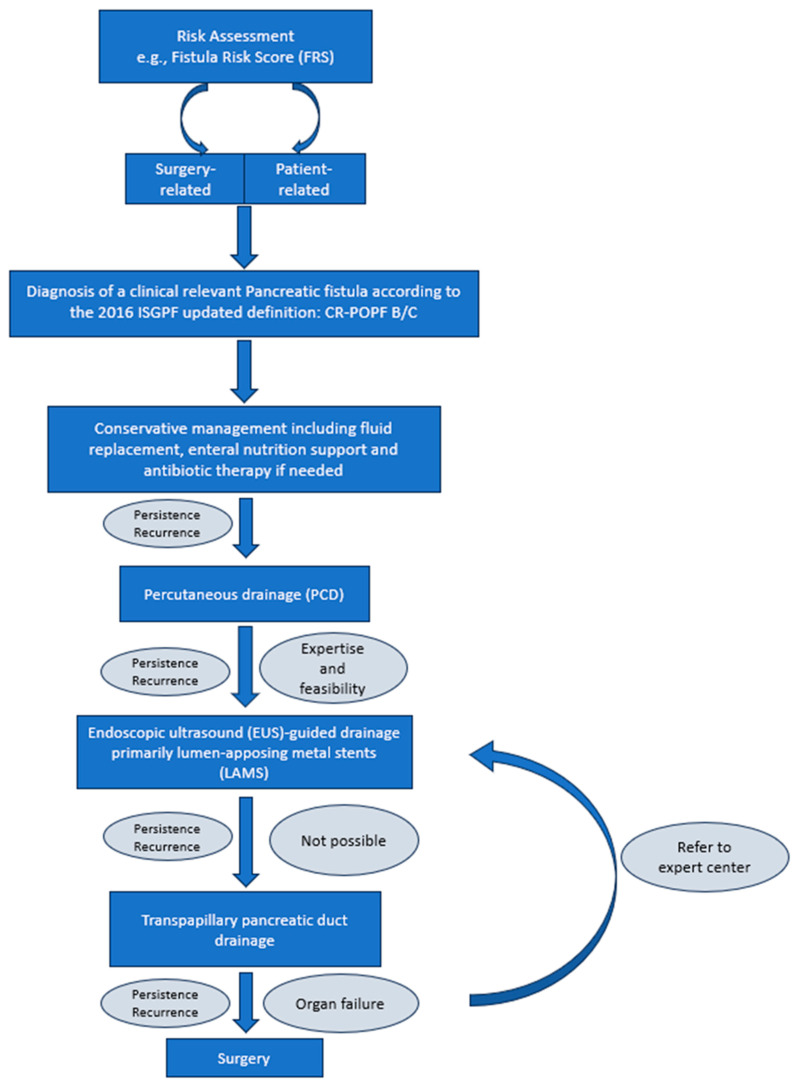
Possible Treatment approach.

Keeping these aspects in mind, we developed a possible algorithm using a step-up treatment approach as shown in Figure 4.

1. Risk assessment (surgery on or near the pancreas, abdominal trauma, severe pancreatitis):
  - Identify patients who are at risk of developing pancreatic fistulas by using suitable tools such as the Fistula Risk Score (FRS)
  - Consider surgery-related factors: non-performance of ligation of the main pancreatic duct, transection at the tail, additional organ resection, complexity of the surgery, texture of the pancreas, etc.
  - Consider patient-related factors: smoking, high BMI, male gender, age, the presence of diabetes mellitus, etc.
2. Diagnosis and severity:
  - Use the Updated International Study Group for Pancreatic Fistula (ISGPF) criteria for diagnosis
  - Classify pancreatic fistulas according to the ISGPF grading system
  - Focus on ISGPF (grades B/C) as they are associated with an extended hospital stay and increased mortality risk
3. Treatment using a step-up approach:
  - Start with conservative management, including operative drain maintenance, adequate intensive care support, enteral nutrition and antibiotic therapy, for patients with organ failure and infection
  - If the fistula persists or recurs:
    - Initiate/Maintain a percutaneous drainage (PCD) and monitor the amount of fluid evacuation
  - If the fistula persists or recurs (tertiary center)
    - Evaluate the feasibility of an endoscopic ultrasound (EUS)-guided drainage, especially the use of a lumen-apposing metal stent (LAMS)
  - If the fistula persists or recurs (low-volume center or technical not possible)
    - Consider transpapillary pancreatic duct drainage
  - Reserve surgical interventions for patients who are not suitable candidates for minimally invasive approaches or whose condition worsens rapidly
  - Consider the use of pharmacological treatments such as somatostatin analogues as an add on, based on individual patient factors
4. Monitoring and follow-up:
  - Assess the amount of fluid evacuation if percutaneous drainage is still in place and remove the catheter as early as possible
  - Monitor fistula closure which may vary depending on the treatment strategy. Look regularly for any potential complications
  - If the fistula persists
    - Consider changing the treatment approach or refer to an expert center
5. Management of complications;
  - Address complications that may arise, such as delayed gastric emptying, abscess formation, or hemorrhage, using appropriate interventions
6. Long-term follow-up.
  - Continue regular follow-up visits to monitor for any recurrence of the fistula or related issues

It is important to note that this algorithm reflects a general approach for managing pancreatic fistulas and should be tailored to each patient’s specific situation. Individual patient characteristics, available resources, and the latest evidence in the field should be considered.

The following flowchart provides a visual representation of the algorithm presented above (Figure 4). The specific details of each step and the decision-making processes may vary based on the clinical context.

Limitations of our study include its retrospective design resulting in a missing systematic approach and the single-center-dependent patient selection. Furthermore, a consequent risk assessment of patients before surgery and intraoperatively was missing. Treatment interventions were based on habits and personal experience. Moreover, looking at the volume of patients and the complex individual patient courses hampering standardization, a prospective randomized trial risks to fail achieving statistically significant differences. 

We conclude that an adequate pre- and intraoperative risk evaluation should be implemented, and preventive options considered in high-risk situations. While endoscopic transmural treatment of persistent or recurrent pancreatic fistulas with LAMS seems to be a safe and effective technique, there is a need to state patient selection and procedural standards within the bundle of treatment strategies for pancreatic fistulas. Finally, a more evidence-based algorithm for the therapy of pancreatic fistulas should be the objective.

## Figures and Tables

**Table 1:** Patient demographics and fistula characteristics (clinically relevant).

	Pancreatic Fistulas (*n* = 60)
Age (years) *	64
Sex, no. (%)	
Female	27 (45)
Male	33 (55)
Pancreatic fistula, etiology *n* (%)	
POPF	46 (76.7)
Acute necrotizing pancreatitis	7 (11.6)
Chronic pancreatitis	4 (6.7)
Abdominal trauma	3 (5)
Updated ISGPF Grading, *n* (%)	
Grade A (biochemical leak)	0 (0)
Grade B	53 (88.3)
Grade C	7 (11.7)

Values in parentheses are percentages unless indicated otherwise; * values are means.

**Table 2:** Indications for surgery leading to clinically relevant POPF.

Histopathologic Findings, *n* (%)	CR-POPF (*n* = 46)	
	POPF Grade B	POPF Grade C
Pancreatic adencoarcinoma	15 (32.7)	2 (4.3)
Pancreatic neuroendocrine tumor	7 (15.2)	2 (4.3)
Duodenal adenocarcinoma	3 (6.5)	0
Gastric cancer	4 (8.7)	1 (2.2)
Cystic tumor	7 (15.2)	0
Ovarian cancer, cytoreductive surgery	3 (6.5)	1 (2.2)
Laparoscopic biopsy	1 (2.2)	0

Values in parentheses are percentages.

**Table 3:** Treatment modalities of clinically relevant pancreatic fistulas.

Treatment Modality	Pancreatic Fistulas (*n* = 60)
Type of drainage ^a^, *n* (%)	
Intraoperatively placed	46 (76.7)
Secondary percutaneous	22 (36.6)
Endoscopic transpapillary	13 (21.7)
Interventional transmural	25 (41.7)
More than one treatment approach	38 (63.3)
Medical treatment *n* (%)	
Somatostatin analogue	1 (1.7)
Treatment of complications ^a^, *n* (%)	
Change of treatment strategy, *n* (%)	38 (63.3)
Need for re-drainage, *n* (%)	5 (8.3)
Reoperation, *n* (%)	3 (5)
Mortality 30d, *n* (%)	4 (6.7)

^a^ Multiple answers possible, values in parentheses are percentages.

**Table 4:** Technical success rate and time until fistula closure for percutaneous and transpapillary drainage.

Clinical Outcome	Percutaneous Drainage Alone (PCD) (*n* = 22)	Transpapillary Drainage + PCD (*n* = 13)	Additional Medical Treatment (*n* = 1)
Technical success rate, *n* (%)	22 (100%)	13 (100)	n/a
Fistula closure rate, *n* (%)	22 (100%)	13 (100)	1
Time until fistula closure in days *	29 (13–58)	n/a	n/a

Values in parentheses are percentages, * values are means.

**Table 5:** Technical success rate, time until fistula closure and mortality rate for transmural drainage.

Clinical Outcome	Interventional Transmural Drainage (*n* = 25)
Technical success rate, *n* (%)	24 (96)
Fistula closure rate, *n* (%)	22 (88)
Time until fistula closure in months *	2.7 (2.1–5.3)
Mortality 30d	2 (8)

Values in parentheses are percentages, * values are means.

## Data Availability

Data supporting reported results can be found at the LAMS data set of the Department of Gastroenterology, Ordensklinikum Linz and the prospective pancreas registry that is conducted by the surgical department, Ordensklinikum Linz.
